# Influence of growth stage on activities of polyhydroxyalkanoate (PHA) polymerase and PHA depolymerase in *Pseudomonas putida *U

**DOI:** 10.1186/1471-2180-10-254

**Published:** 2010-10-11

**Authors:** Qun Ren, Guy de Roo, Bernard Witholt, Manfred Zinn, Linda Thöny-Meyer

**Affiliations:** 1 Laboratory for Biomaterials, Swiss Federal Laboratories for Materials Science and Technology (Empa), CH-9014 St. Gallen, Switzerland; 2 Synthon BV, P.O. BOX 7071, 6503 GN Nijmegen, the Netherlands; 3 Institute of Molecular Systems Biology, Swiss Federal Institute of Technology, CH-8093 Zurich, Switzerland

## Abstract

**Background:**

Medium chain length (mcl-) polyhydroxyalkanoates (PHA) are synthesized by many bacteria in the cytoplasm as storage compounds for energy and carbon. The key enzymes for PHA metabolism are PHA polymerase (PhaC) and depolymerase (PhaZ). Little is known of how mcl-PHA accumulation and degradation are controlled. It has been suggested that overall PHA metabolism is regulated by the β-oxidation pathway of which the flux is governed by intracellular ratios of [NADH]/[NAD] and [acetyl-CoA]/[CoA]. Another level of control could relate to modulation of the activities of PhaC and PhaZ. In order to investigate the latter, assays for *in vitro *activity measurements of PhaC and PhaZ in crude cell extracts are necessary.

**Results:**

Two *in vitro *assays were developed which allow the measurement of PhaC and PhaZ activities in crude cell extracts of *Pseudomonas putida *U. Using the assays, it was demonstrated that the activity of PhaC decreased 5-fold upon exponential growth on nitrogen limited medium and octanoate. In contrast, the activity of PhaZ increased only 1.5-fold during growth. One reason for the changes in the enzymatic activity of PhaC and PhaZ could relate to a change in interaction with the phasin surface proteins on the PHA granule. SDS-PAGE analysis of isolated PHA granules demonstrated that during growth, the ratio of [phasins]/[PHA] decreased. In addition, it was found that after eliminating phasins (PhaF and PhaI) from the granules PhaC activity decreased further.

**Conclusion:**

Using the assays developed in this study, we followed the enzymatic activities of PhaC and PhaZ during growth and correlated them to the amount of phasins on the PHA granules. It was found that in *P. putida *PhaC and PhaZ are concomitantly active, resulting in parallel synthesis and degradation of PHA. Moreover PhaC activity was found to be decreased, whereas PhaZ activity increased during growth. Availability of phasins on PHA granules affected the activity of PhaC.

## Background

Polyhydroxyalkanoates (PHA) are intracellular carbon storage polyesters that are produced by a wide variety of bacteria [1]. The most common PHA variants are so-called short chain length (scl-) PHAs containing monomers with 4 and/or 5 carbon-atoms [1]. Most other PHAs are referred to as medium chain length (mcl-) PHAs because the monomers generally consist of 3-hydroxyalkanoic acids with 6 or more C-atoms [2]. These mcl-PHAs which are produced by fluorescent pseudomonads have application potential as elastomeric biodegradable plastics [3] or as sources of chiral monomers [4-6].

*Pseudomonas putida *accumulates mcl-PHA in discrete granules covered by a phospholipid monolayer in which various proteins are embedded [7,8]. These granule-associated proteins include PHA polymerases (PhaC), PHA depolymerase (PhaZ) [9-11], phasins (PhaF and PhaI) [7,12,13] and acyl-CoA synthetase [14]. PHA polymerases (also referred to as PHA synthases), which use CoA-activated 3-hydroxy fatty acids as substrates, are the key enzymes in mcl-PHA biosynthesis [15]. In *P. putida *U, two PHA polymerases encoded by *phaC*1 and *phaC*2 are known [16]. Disruption of *phaC*2 appeared to reduce the accumulation of PHA by two thirds, whereas disruption of *phaC*1 resulted in a complete loss of PHA accumulation [16]. Intracellular mcl-PHA degradation proceeds through the action of a PHA depolymerase encoded by *pha*Z. The enzyme has been suggested to act via an exo-acting hydrolytic mechanism [17]. The major amount of granule associated proteins in *P. putida *is accounted for by the phasins PhaI and PhaF [12,13]. These amphiphilic proteins undoubtedly have a structural role in the granule, by which a barrier is created between the hydrophobic surface of the polymer and the surrounding hydrophilic cytoplasm [18]. In addition, PhaF may also regulate PHA metabolism at the transcriptional level [13].

Little is known of how mcl-PHA accumulation and degradation are controlled in pseudomonads. Previous studies have demonstrated that in *P. putida*, PHA polymerases and PHA depolymerase are concomitantly active, resulting in parallel synthesis and degradation [19]. Although this would generate a futile cycle, it has been suggested that overall PHA metabolism is regulated by the β-oxidation pathway whereby the flux is governed by intracellular ratios of [NADH]/[NAD] and [acetyl-CoA]/[CoA] [19,20].

Another level of control could relate to modulation of the specific activities of PhaC and PhaZ. In order to investigate this possibility, two assays were developed which enable *in vitro *activity measurements of PhaC and PhaZ in crude cell extracts of *P. putida *U. Using these assays, we followed the activities of PhaC and PhaZ during growth and correlated these to the amount of phasins on the PHA granules.

## Results

### Development of an *in vitro *activity assay for measuring PHA polymerase (PhaC) activity in crude cell extracts

Up to now, few studies have reported on the enzymology and physiology of mcl-PHA polymerases. This is due to the difficulty of purifying an active mcl-PHA polymerase and the lack of an efficient *in vitro *activity assay for mcl-PHA polymerases. We have developed an *in vitro *PhaC activity assay for granule-associated PhaC activity [21]. This assay is, however, not suitable for measuring activity in crude cell extracts, due to the strong background caused by thioesterases which compete for the PhaC substrate.

**Figure 1:**
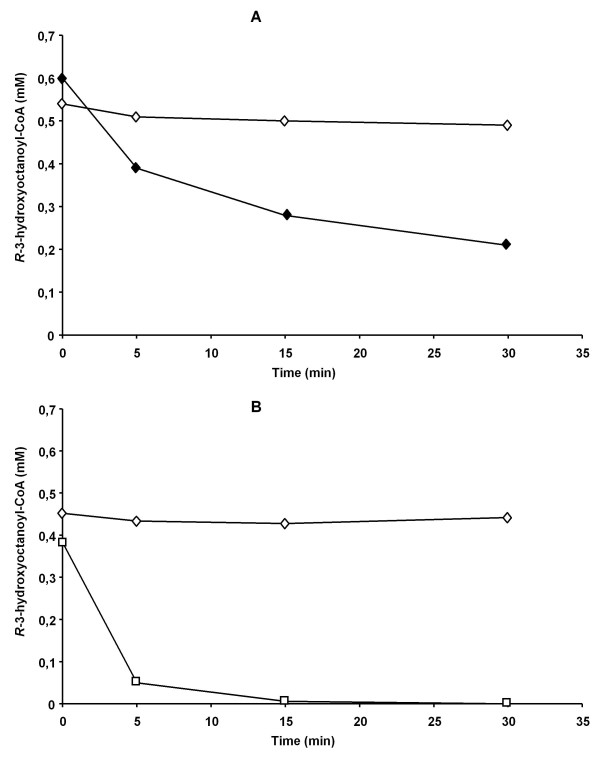
**Consumption of *R-*3-hydroxyoctanoyl-CoA in crude cell extracts of *P. putida *U and *P. putida *U::*pha*C1^-^**. Panel A: Influence of free CoA on *R-*3-hydroxyoctanoyl-CoA thioesterase activity in a crude cell extract of *P. putida *U::*pha*C1^-^. Assay conditions: 100 mM Tris-HCl, pH 8, 1 mg/ml BSA, 0.5 mM MgCl_2_, 0.5 mM *R-*3-hydroxyoctanoyl-CoA, 0.1 mg/ml crude cell extract of *P. putida *U::*pha*C1^-^ with no CoA (filled diamond) or 1 mM CoA (open diamond). Data represent the average of two measurements. Panel B: *R-*3-hydroxyoctanoyl-CoA consumption in crude cell extracts of *P. putida *U::*pha*C1^-^ and *P. putida *U in the presence of free CoA. Assay conditions: 100 mM Tris-HCl, pH 8, 1 mg/ml BSA, 0.5 mM MgCl_2_, 0.5 mM *R-*3-hydroxyoctanoyl-CoA, 1 mM CoA, 4 mg/ml crude cell extract of either *P. putida *U::*pha*C1^-^ (open diamond) or *P. putida *U (open square). *R-*3-hydroxyoctanoyl-CoA depletion was measured by HPLC. Data represent the average of two measurements.

An improved assay was developed in which thioesterases activity is suppressed by addition of free CoA. This is illustrated in Figure 1A in which a crude extract of a polymerase knock-out mutant *P. putida *U::PhaC1^-^ was used. This mutant was found to grow well on fatty acids but was unable to produce PHA. Due to the presence of interfering acyl-CoA thioesterases in the extract, *R*-3-hydroxyoctanoyl-CoA was rapidly depleted. However, addition of CoA reduced the consumption of acyl-CoA by 90%, probably due to product inhibition of the thioesterases [22]. Although PhaC itself is known to be slightly inhibited by free CoA, with a *K*i of 0.715 mM [23], the assay permitted measuring PhaC activity in crude cell extracts. This was demonstrated by comparison of the rate of *R*-3-hydroxyoctanoyl-CoA consumption by crude extracts of *P. putida *U and *P. putida *U::PhaC1^-^ (Figure 1B).

The only difference between the strains is the presence of a functional PHA polymerase in *P. putida *U. Therefore, the difference in consumption of *R*-3-hydroxyoctanoyl-CoA between the PhaC1^-^ and PhaC1^+ ^strains must be due to the activity of PhaC1. Based on the measurements, an activity of 23.4 U/g total proteins was calculated. In *P. putida *GPo1, the amount of PhaC1 was estimated to account for 0.075% of total cellular protein [24]. Using this estimate and by assuming that only PhaC1 was expressed and PhaC2 not expressed, a specific activity of 31.2 U/mg PhaC1 was calculated. This activity was in the same range as found for polymerase bound to isolated PHA granules [23].

### Development of an *in vitro *activity assay for measuring PHA depolymerase (PhaZ) activity in crude cell extracts

**Figure 2:**
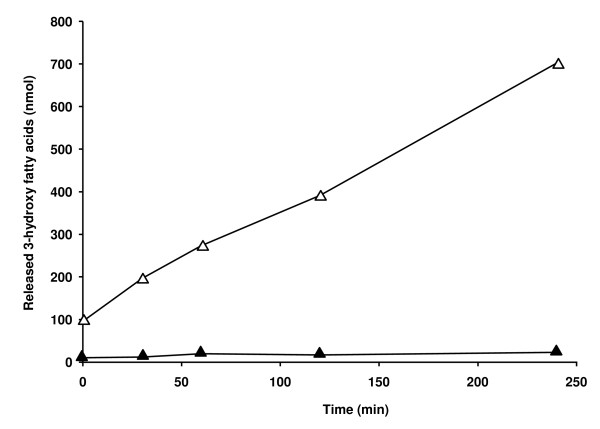
**Production of 3-hydroxyalkanoic acid in crude cell extracts of *P. putida *U and *P. putida *U::*pha*Z^-^**. Cells grown to the stationary phase (16 h in 0.2NE2 medium + 15 mM octanoate) were harvested, resuspended to 1 mg total protein/ml in 100 mM Tris-HCl, pH 8, 0.5 mM MgCl_2_, and lysed by three passages through a French pressure cell. The production of PHA monomers was followed for *P. putida *U::*pha*Z^-^ (filled triangle) and *P. putida *U (open triangle). Supernatants (250 μl) containing 3-hydroxyalkanoic acids were lyophilyzed and methanolyzed prior to analysis by GC. Data represent the average of two measurements.

Similar to PHA polymerases, characterization of intracellular mcl-PHA depolymerases (PhaZ) under different physiological conditions has been hampered due to the lack of a suitable *in vitro *activity assay that can be used in crude cell extracts. An easy assay for determining PhaZ activity has been reported by monitoring the pH changes caused by the release of 3-hydroxy fatty acid monomers [25], however, it is only suitable for depolymerase activity measurements from purified PHA granules. Here, a depolymerase assay was developed in which the release of 3-hydroxy fatty acid monomers is quantified directly. The released monomers were separated from the insoluble polymer and other cell material by centrifugation and were subsequently methanolyzed to yield volatile methyl-esters which was measured by GC analysis. Upon incubation of a crude extract of *P. putida *U (which had been grown on octanoate) in Tris-HCl buffer, almost linear increases of 3-hydroxyoctanoate, and to a minor extent 3-hydroxyhexanoate, were observed. Figure 2 shows the total amount of 3-hydroxy fatty acids released over time.

No increase was observed when a crude extract of *P. putida *U::PhaZ^-^ (disrupted in *pha*Z) was used, thus indicating that PhaZ accounts for the production of 3-hydroxy fatty acids. An activity of 10 U/g total proteins could be calculated.

### Growth stage dependent activities of PhaC and PhaZ

**Figure 3:**
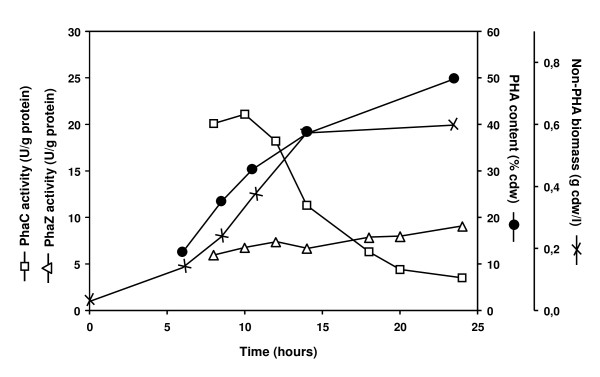
**Enzymatic activities of PhaC and PhaZ during growth of *P. putida *U on octanoate**. *P. putida *U was grown on 15 mM octanoate in nitrogen limited medium (0.2 NE2). Culture aliquots were harvested, resuspended to 1 mg total protein/ml and lysed by three passages through a French pressure cell and analyzed for non-PHA biomass (x, right scale), accumulation of mcl-PHA relative to the total cell dry weight (cdw) (filled circle, right scale), activities of PhaC (open square, left scale) and PhaZ (open triangle, left scale). Data represent the average of two measurements.

Using the newly developed assays, the activities of both PhaC and PhaZ in different growth stages were investigated. *P. putida *U was grown on octanoate, cells were harvested every 2 hours and analyzed for biomass, PHA content and PhaC and PhaZ activities (Figure 3).

**Figure 4:**
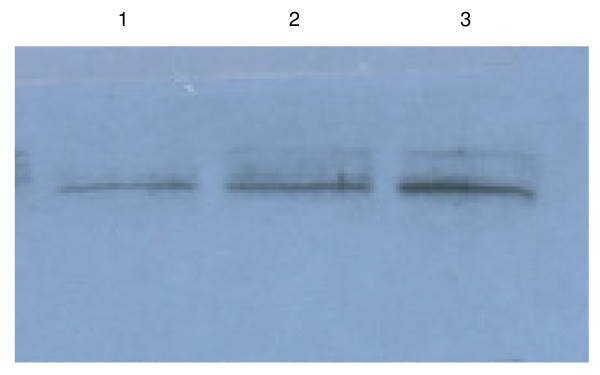
**Western blot analysis of PhaC1 in *P. putida *U harvested at different growth stages**. *P. putida *U was grown on 15 mM octanoate in nitrogen limited medium (0.2 NE2). Antibodies specific against PhaC1 were used to follow PhaC1 levels in *P. putida *U cells grown on octanoate and harvested after 8 (lane 1), 14 (lane 2) and 25 hours (lane 3). All lanes were loaded with an equal amount of cellular protein (20 μg).

Cell cultures reached a maximum biomass of 1.3 g/l with a maximum PHA content of 49% relative to the total cell dry weight. By substraction of the amount of PHA from the total amount of biomass, the residual biomass was calculated. High PhaC activity was found in the early growth stages with a maximum of 21 U/g total proteins. Surprisingly, PhaC activity decreased at least 5-fold during growth, reaching an activity of only 6 U/g total protein in the early/mid stationary growth phase, and 4 U/g total protein in the late stationary growth phase. Western blot analysis using specific anti-PhaC1 antibodies demonstrated that the decrease in PhaC activity is not due to a decrease of expression of PhaC. In fact, the cellular amount of PhaC increased slightly during growth (Figure 4). Therefore, it is very likely that during exponential growth, the specific activity of PhaC (in U/mg PhaC) is reduced dramatically.

In contrast to PhaC, the PhaZ activity increased slightly during growth with values varying from 5-10 U/g total proteins. PhaZ activity was already obvious in the very early stages of PHA accumulation (i.e 5.5 U/g total proteins in the early exponential growth phase). PhaZ could not be detected in crude cell extracts due to the lack of a sensitive anti-PhaZ antibody. Thus, the specific activity could not be estimated.

**Figure 5:**
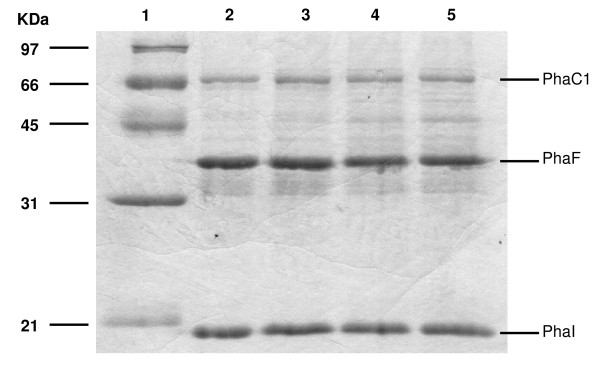
**SDS-PAGE analysis of PHA granules isolated in different growth phases**. Lanes: Molecular weight marker (kD, lane 1), PHA granules isolated from *P. putida *U after 8 hours (lane 2), 14 hours (lane 3), 20 hours (lane 4) and 25 hours (lane 5) of growth on octanoate. Increasing amounts of PHA granules were applied: 0.1 mg (lane 2), 0.5 mg (lane 3), 1 mg (lane 4) and 1.5 mg (lane 5), respectively. Experiments were performed three times. For different cultivations, the absolute values regarding total amount of PHA granule-attached proteins had variations due to sample taken at different time points; however, PHA reganule-attached proteins exhibited similar pattern relative to cell growth in these three experiments. In this study, only the results obtained from one experiment were presented.

To understand the observed decrease of PhaC activities and increase of PhaZ activities, PHA granules were isolated from *P. putida *U after 8, 14, 20 and 25 hours of growth on octanoate. All four granule preparations were analyzed by SDS-PAGE in order to see differences in protein composition (Figure 5). No significant changes could be observed between the different granule preparations, except that the amount of the phasin PhaF was slightly decreased after 14 hours. When the amount of PHA granules which were loaded on the SDS-polyacrylamide gel was taken into account, it appeared that isolated granules harvested after 25 hours of growth contained much less proteins as compared to PHA granules harvested after 8 hours. This indicated that PHA granules harvested at a later growth stage had smaller surface areas for protein binding. Furthermore, there was an increased background of "contaminating" proteins at later growth stages (Figure 5), possibly caused by non-specific binding to the PHA surface [26].

### Effect of phasins on PhaC activity

One of the possibilities for the decrease in activity of PhaC and increase in activity of PhaZ could relate to changes in the amounts of available phasins on the PHA granule. In order to examine this hypothesis we used a *P. putida *mutant which is deficient in both PhaI and PhaF phasins. Both the wild type and mutant strains were grown on octanoate for 10 hours before PHA granules were isolated. Table 1 lists PhaC activities of PHA granules isolated from different *P. putida *strains together with the corresponding mutants.

**Table 1:** Granule-bound PhaC activities of various *P. putida *mutants

Strain	Reference	PHA granule phasins		Granule-bound PhaC activity (U/mg PhaC)
		**PhaF**	**PhaI**	

*P. putida *U	[16]	+	+	40.2
*P. putida*::*phaZ*^-^	[16]	+	+	44.9
*P. putida *BMO1	[32]	+	+	42.2
*P. putida *BMO1-42	[32]	-	-	12.7
*P. putida *GPo1	[15,23]	+	+	42.3
*P. putida *GPG-Tc-6	[13,23]	-	+	38.0
*P. putida *GPo1001	[31,23]	+	-	29.5

Assay conditions: 100 mM Tris-HCl, pH 8, 1 mg/ml BSA, 0.5 mM MgCl_2_, 0.0125-0.25 mM *R-*3-hydroxyoctanoyl-CoA and 0.2 μg/ml granule-bound PhaC (granules isolated after growth for 10 hours). Initial activity was measured spectrophotometrically (*A*412) by following release of CoA using DTNB. PhaC amounts were estimated by densitometric scanning of SDS-polyacrylamide gels.

The PhaC activity on granules of *P. putida *BMO1 42 (Δ*phaI*, Δ*phaF*) was found to be 3-fold lower than that of granules isolated from the wild type *P. putida *BMO1 and *P. putida *U. Since this mutant lacked both PhaI and PhaF, it is likely that the presence of these phasins stimulates PhaC activity. Previously, we have reported that PhaF^-^ granules of *P. putida *GPG-Tc6 did not show a significant reduction of activity as compared to granules from the parental strain *P. putida *GPo1 [23], whereas, a 1.5-fold reduction of PhaC activity could be demonstrated for PhaI^-^ granules of *P. putida *GPo1001 [23]. These results indicate that PhaI has more impact on PhaC activity than PhaF. Yet, the highest impact is observed when both phasins are absent. The influence of PhaF and PhaI on the specific activity of PhaZ could not be investigated due to lack of accuracy in determining the amount of granule-associated PhaZ.

## Discussion

Two activity assays were developed which allow rapid measurements of PHA polymerases and PHA depolymerases in crude extracts from cells harvested at different growth stages (Figures 1 and 2). Using these assays with whole cell lysates, we demonstrated a 5-fold decrease in the activity of PhaC and a 1.5-fold increase in the activity of PhaZ during exponential to stationary phase growth of *P. putida *U on octanoate (Figure 3). These results were consistent with the *in vitro *activity studies using isolated PHA granules harvested at different growth stages [23]. The results obtained here also confirm previous data in which parallel PHA accumulation and degradation was demonstrated [19,27].

Regarding the decrease of PhaC activity with the growth of bacteria, previously we have shown that the PhaC activity is influenced by the physiological stage of the cells: the activity of PhaC is stimulated by the high ratio of [3-hydroxyacyl-CoA]/[CoA] [19]. It is likely that at the beginning of the growth phase (high growth rate), CoA and NAD^+ ^are consumed, and acetyl-CoA and NADH are produced via β-oxidation for growth, leading to high ratios of [acetyl-CoA]/[CoA] and [NADH]/[NAD], which further resulting in high ratio of [3-hydroxyacyl-CoA]/[CoA] [19], thus, higher activity of PhaC. In contrast, when cells enter the stationary growth phase, β-oxidation is not highly active anymore, the ratios of [acetyl-CoA]/[CoA] and [NADH]/[NAD] are likely to decrease, leading to lower ratio of [3-hydroxyacyl-CoA]/[CoA] [19], thus lower activity of PhaC. Therefore, even through PhaC content was increased with the growth of bacteria (Figure 4), the activity of PhaC was decreased (Figure 3). In addition to the effect of physiological reagents on PhaC activity, in this study, we further investigated the influence of phasins and found that availability of both PhaI and PhaF have significant impact the activity of PhaC (Table 1).

Although the PHA granules became larger as the culture aged [28,29], this was not associated with an increase of the amount of phasins (Figure 5). The availability of phasins could be one of the reasons for the observed changes in enzyme activities of PhaC. At the initial accumulation stage, young PHA granules may be fully covered with phospholipids and proteins. Interactions between the enzymes and granule-bound phasins may be important for optimal polymerase activity because in the absence of phasins the specific PHA polymerase activity was reduced (Table 1). The polymerases produce PHA continuously, allowing the granules to grow as the culture proceeds from the exponential to the early stationary growth phase. The data of Figures 3 and 5 show that the granule attached proteins do not keep pace with the total amount of PHA produced thus indicating a reduction in the ratio of protein to PHA on these granules. As the very hydrophobic PHA presumably does not remain exposed directly to the aqueous cytoplasm, lipids and proteins with significant hydrophobic surfaces will likely bind to such exposed PHA surface. As a result, there might be non-specific binding of proteins to the granule surface of older PHA granules. Evidence that this phenomenon occurs is the 5 - 15 fold reduced ratio of the amount of phasins *versus *granule mass and the increased number of non-specific proteins which bind to PHA granules as the culture ages (Figure 5).

Although not essential for PHA synthesis [19,30], phasins dramatically affect PHA accumulation as has been demonstrated for various *Pseudomonas *disruption mutants [23,31,32]. Detailed analysis of the interactions between PhaC/PhaZ and phasins as well as disruption mutants of phasins will be required for further insight in the physiological relevance of phasins. The newly described PhaZ and PhaC assays could be useful tools for such investigations.

## Conclusions

Although molecular analysis of mcl-PHA polymerase and depolymerase has provided information on catalytic mechanisms (see review [8]), much research still has to be undertaken at the biochemical level of these enzymes. Here we describe the development of activity assays for PhaC and PhaZ allowing their use in crude cell extracts. We followed the activities of these two enzymes during growth and found that in *P. putida *PhaC and PhaZ are concomitantly active, resulting in parallel synthesis and degradation. It was also found that PhaC activity was decreased significantly towards the beginning of the stationary growth phase, whereas PhaZ activity was increased slightly from exponential growth to stationary growth phase. Moreover, availability of phasins on PHA granules has an impact on the activity of PhaC.

## Methods

### Materials

*R/S-*3-hydroxyalkanoic acids were supplied by Sigma (St. Louis, US). *R*-3-hydroxyoctanoic acid was prepared via hydrolysis of mcl-PHA [4]. *R*-3-hydroxyoctanoyl-CoA was synthesized as described previously [21]. The concentration of *R*-3-hydroxyoctanoyl-CoA was estimated by hydroxylamine treatment [33], which causes the release of bound CoA. The concentration of free CoA before and after hydroxylamine treatment was determined with the Ellman method [34].

### Bacterial strains

*P. putida *U, *P. putida *U::*pha*C1^-^, *and P. putida *U::*pha*Z^-^[16] were kindly provided by Prof. J. M. Luengo (University of Leon, Spain). *P. putida *BMO1 (wild type) and *P. putida *BMO1 42 (Δ*phaI*, Δ*phaF*) [32] were kindly provided by Dr. H. Valentin (Monsanto, U.S). All strains including *P. putida *GPo1 [15], *P. putida *GPG-Tc6 (Δ*phaF*) [13] and *P. putida *GPo1001 (Δ*phaD*) [31] were precultured on Luria-Bertani medium. In order to stimulate PHA accumulation, all *Pseudomonas *strains were cultivated in 0.2 NE2 medium (mineral medium containing 20% of the total nitrogen of E2 medium) supplemented with 15 mM sodium octanoate [35]. Cells were harvested at different cultivation times and stored in small batches at -20°C.

### PHA granule isolation and analysis of granule-associated proteins

PHA granules of *P. putida *were isolated from the cells by density centrifugation as previously reported [21]. Cells were resuspended in H_2_O to a final concentration of 50 mg/ml and disrupted by three passages through a pre-cooled French pressure cell. Broken cells (50 mg/ml) (30 ml) were loaded on top of a 20% sucrose layer (200 ml) and subsequently centrifuged (15,000 g) for 3 hours. The PHA granules, which remained on top of the sucrose layer, were collected and washed twice with 100 mM Tris-HCl pH 8. The final PHA pellet was resuspended in 30 ml of 100 mM Tris-HCl pH 8. Samples of purified granules were mixed 1:1 (v/v) with SDS-loading buffer [36] and the bound proteins were separated on SDS-polyacrylamide gels as described before [37]. PHA polymerase amounts were estimated by densitometric scanning of SDS-polyacrylamide gels using a Multimage™ Light Cabinet (Alpha Innovation Corp.) with chemiluminescence and visible light imaging. Protein bands from various purification fractions were compared to protein bands of known amounts of BSA. Released proteins from PHA granules were quantified with Bradford assay using BSA as the standard [38].

### PHA polymerase (PhaC) activity assay

PHA polymerase activity was analyzed by following the release of CoA using DTNB. A typical mixture (300 μl) contained 0.5 mM *R*-3-hydroxyoctanoyl-CoA, 0.1-1 mg/ml PHA granules, 1 mg/ml BSA, 0.5 mM MgCl_2 _in 100 mM Tris-HCl, pH 8. Activity was measured spectrophotometrically as previously described [21]. PHA polymerase activity in crude cell extract was measured by following the depletion of *R-*3-hydroxyoctanoyl-CoA using HPLC [39]. A typical reaction mixture contained 0.5 mM *R-*3-hydroxyoctanoyl-CoA, 1 mM CoA, crude cell extract (0.1 - 4 mg total protein/ml), 1 mg/ml BSA and 0.5 mM MgCl_2 _in 100 mM Tris-HCl, pH 8. One unit is defined as 1 μmol *R*-3-hydroxyoctanoyl-CoA consumption per minute. Values presented here are the average of two determinations.

### PHA depolymerase (PhaZ) activity assay

PHA depolymerase activity was analyzed by following the release of 3-hydroxyacid monomers by gas chromatography (GC). A typical mixture (2 ml) contained crude cell extract of *P. putida *U (1 mg total protein/ml) and 0.5 mM MgCl_2 _in 100 mM Tris-HCl pH 8. Aliquots (250 μl) were taken at timed intervals and the reaction stopped by the addition of 250 μl ice-cold ethanol. After pelleting of the precipitated proteins and granules by centrifugation (20,000 rpm, 30 min), supernatant (400 μl) was transferred to a pyrex tube and subsequently lyophilized. The lyophilized samples containing the released 3-hydroxyacids were methanolyzed by addition of 1 ml chloroform and 1 ml acidified methanol (containing 15% H_2_SO_4_), followed by heating in an oil bath (100°C, 2.5 hours). Addition of 1 ml H_2_O and subsequent thorough shaking resulted in the separation of two phases. The upper phase (methanol, H_2_O and H_2_SO_4_) was discarded. The lower phase (containing the 3-hydroxyacyl methylesters) was dried over Na_2_SO_4 _and analyzed by GC.  One unit is defined as 1 μmol *R*-3-hydroxyoctanoic acid production per minute. Values presented here are averages of two determinations.

### Expression and purification of PhaC1 from *P. putida *U for preparation of anti-PhaC1 antibodies

Purification of PhaC1 was achieved by using N-terminal His6-tag fusions. Two degenerate primers (BamH1 5' GTGGATCCGTAACAAGAACAACGATGAGCTGCAGCGGC 3' and *Xba*I 5' CTGTCTAGAAAAAAGTCCCGTGGCGCTC 3') were used to amplify *phaC*1 from *P. putida *U. The amplified gene was cloned into pKB-2, digested with *Bam*H1/*Sac*I and cloned into the commercial vector pQE-32 (Qiagen). After overexpression of *phaC*1 in *E. coli *XL-Blue, PhaC1 was purified by metal chelate affinity chromatography (Qiagen). Antibodies against purified PhaC1 were prepared as previously described [40].

## List of abbreviations

PHA: Polyhydroxyalkanoate; Mcl: Medium chain length; PhaC: PHA polymerase; PhaZ: PHA depolymerase; BSA: Bovine Serum Albumin; CoA: Coenzyme A; DTNB: 5,5'-Dithiobis(2-nitrobenzoic acid); GC: Gas Chromatography; HPLC: High Performance Liquid Chromatography; SDS-PAGE: Sodium Dodecyl Sulfate Poly Acrylamide Gel Electrophoresis.

## Competing interests

The authors declare that they have no competing interests.

## Authors' contributions

QR and GdR performed the laboratory experiments and drafted the manuscript. BW advised the experimental design and revised the drafted manuscript. MZ and LTM helped in preparing of the manuscript. All authors read and approved the final manuscript.
